# Microglial heterogeneity after subarachnoid haemorrhage

**DOI:** 10.1002/ctd2.61

**Published:** 2022-05-01

**Authors:** Ian Galea

**Keywords:** subarachnoid haemorrhage, microglia, single-cell transcriptomics, single-cell RNA-seq, haemoglobin, brain

A specific type of haemorrhagic stroke called aneurysmal subarachnoid haemorrhage (SAH) affects people of working age and is more costly than other types of strokes. After SAH, people may look outwardly normal but experience a range of neurocognitive deficits ^1^that affect their quality of life and ability to return to full productive work. SAH is caused by rupture of an aneurysm, which is a bulging structural abnormality of the wall of a cerebral artery in the subarachnoid space, on the brain surface. This results in an injection of blood into the subarachnoid space under arterial pressure with a number of pathological consequences which may in turn affect clinical outcome. Immediate effects include mechanical injury and ischaemia (since increased intracranial pressure reducescerebral perfusion within the rigid confines of the skull). Delayed effects include inflammation, the toxic effects of blood-derived products such as haemoglobin, vasospasm and hydrocephalus.

Microglia are the brain’s resident tissue macrophages, constituting circa 10% of the total cellular population.^2^ They are derived from the yolk-sac and self-renew^2^, so are developmentally and functionally distinct from two other types of myeloid-lineage cells in the intracranial compartment which are replenished continuously from the bloodstream: (1) CD163-positive perivascular, meningeal and choroid plexus macrophages, and (2) monocytes in the cerebrospinal fluid – both of these two populations can migrate into the central nervous system parenchyma during pathology to spatially co-exist with microglia. Microglia can be distinguished from these other types of myeloid-lineage cells by a variety of cell surface markers, which enables one to study them in more detail.

When resting microglia are activated there is a spectacular change in morphology from a highly ramified state to a rounded amoeboid form. While early studies suggested the presence of two types of activated microglia, pro-inflammatory M1 and anti-inflammatory M2, these are now believed to be the opposite ends of what is a wide spectrum of functional specialization of microglia.It is reasonable to hypothesize that microglia after SAH are likely to be functionally heterogenous. First, the different pathological consequences of SAH mentioned above are likely to elicit different microglial reactions. Second,there is a temporal variation in microglial activationrelative to the SAH event, with evidence of a wave of response spreading centrifugally from the base to the cortex of both hemispheres ^3^and a shift from M1 to M2 with time. ^4^Third the microglial reaction is most intense close to the blood clot ^5^or aneurysmal rupture^3, 4^ in both human^3, 5^ and experimental^4^ studies. In summary, microglia after SAH are likely to exhibit functional heterogeneity, with at least three factors determining the functional state of single microglial cells: time post SAH, location (distance away from aneurysm or blood clot) and pathology (ischaemia, mechanical tissue stretch, inflammation, haemoglobin exposure).

Recent work has started to eludicate the functional heterogeneity of microglia after SAH. Bulk transcriptomic studies, during which microglial cells are isolated from the brain and their messenger RNA analysed together, results in a dataset which represents the average state of all microglial cells. On the other hand, more recent technology enables single-cell transcriptomic profiling of microglia and therefore the acquisition of unprecedented detail on microglial heterogeneity. Principal component analysis is then used to group cells with similar transcriptomes into clusters. After induction of experimental SAH using endovascular perforation at the junction between anterior and middle cerebral arteries in mice, Chen et al used single-cell RNA sequencing to studymicroglial heterogeneity after SAH. ^6^Microglia were identified on the basis of their expression of CD11b, Tmem119 and Cx3R1, and isolated for analysis on day three after SAH, at a time point when microglia in this model are undergoing a switch from a M1-predominant to a M2-predominant state,^4^ probably with the intention of capturing maximum microglial heterogeneity. Ten clusters of microglia were identified, of which three were clearly distinct. The differentially expressed genes related to these three clusters were found to be highly expressed when SAH microglia were compared to microglia from control animals, hence validating the existence of these clusters. Two of the three microglial clusters were associated with expression of genes related to inflammation and proliferation while a third cluster expressed a unique set of genes which are openly provided by the authors and will be of great interest to SAH investigators worldwide.

Heterogeneity in the microglial transcriptome is likely to translate into functional heterogeneity. In this respect it is important to contextualize the results of this single-cell RNA-seq study within data in the literature showing that microglia after SAH can have opposing deleterious^3^ and beneficial ^7^effects. As an example of a deleterious effect of microglia, Schneider et al used the endovascular model of experimental SAH in mice to demonstrate microglial accumulation which shared a temporo-spatial profile with neuroaxonal damage as determined by NeuN/TUNEL co-positivityand amyloid precursor protein staining. ^3^Schneider et al depleted microglia in CD11b HSVTK+/- mice (which harbor the HSVTK suicide gene in myeloid cells, imparting sensitivity to ganciclovir) using intraventricular infusion of ganciclovir, and in these animals neuroprotection was observed after experimental SAH, compared to wild-type (CD11b HSVTK−/−) littermates. In this experimental set-up, it was not clear when the ganciclovir started to take effect within the first five days post-SAH, and therefore whether M1 or M2 microglia were predominantly affected. As an example of a beneficial effect of microglia, Shallner et al used a prechiasmatic injection of autologous blood to model SAH in mice and showedthat brain-resident microglia protected against neuronal cell death, vasospasm, and cognitive dysfunction, and improved erythrophagocytosis, via expression of heme oxygenase-1, the inducible isoform of heme oxygenase.^7^ Interestingly the gene for heme oxygenase-1, *Hmox1*, was found to be upregulated in one of the distinct clusters of SAH-associated microglia in the single-cell transcriptomic study referred to above^6^ alongside cytokine, chemokine and other genes defining thispro-inflammatory microglial cluster, perhaps indicating that further subgroups of activated microglial clusters may exist.

What are microglia responding to after SAH?Since the aneurysm and the blood clot are in the subarachnoid space, it is very crucial to recognize the importance of a microglial reaction after SAH, which occurs in the parenchyma not the subarachnoid space – its presence suggestsone of two possibilities: (1) either the occurrence of events within the parenchyma secondary to the SAH (such as ischaemia from vasospasm) or else (2) an intraparenchymal reaction to factors outside the parenchyma. With respect to the latter, a recent post-mortem study of brain tissue from 39 patients dying after SAH and 22 controlsshowed that microglia within the parenchyma upregulated markers of phagocytosis and motility after SAH.^5^ This microglial reaction was highest in the outer cortex closest to the brain surface, with a gradient that diminished inwards in the deeper cortex, in keeping with effects of substances diffusing into the cortex from the subarachnoid space.^5^ The most abundant substance released by the blood clot is haemoglobin, which is toxic to neurones and pro-inflammatory.^8^ Hence efforts to keep haemoglobin out of the parenchyma, by cerebrospinal fluid diversion or by therapeutic strategies that increase subarachnoid haptoglobin concentration, or both, are likely to be of clinical benefit.

Future single-cell transcriptomic studies should study the time course of microglial heterogeneity after experimental SAH. In addition, human studies are needed since there may be substantial differences between rodents and man. Rodent models of SAH tend to underestimate the effect of blood products since cerebrospinal fluidflow rates are higher and intracranial blood clotis cleared more rapidly in rodents. Moreover single-cell transcriptomic studies are starting to reveal profound differences between mouse and man in aging^9^ and disease. ^10^As illustrated by Chen et al, ^6^such studies may lead to discovery of novel post-SAH microglial transcriptional signatures, which in turn may identify novel therapeutic targets.
